# Are stakeholders ready to transform phosphorus use in food systems? A transdisciplinary study in a livestock intensive system

**DOI:** 10.1016/j.envsci.2022.01.011

**Published:** 2022-05

**Authors:** Julia Martin-Ortega, Shane A. Rothwell, Aine Anderson, Murat Okumah, Christopher Lyon, Erin Sherry, Christopher Johnston, Paul J.A. Withers, Donnacha G. Doody

**Affiliations:** a Sustainability Research Institute, School of Earth and Environment, University of Leeds, Leeds, United Kingdom; b Lancaster Environment Centre, Lancaster University, Lancaster, United Kingdom; c School of Chemistry and Chemical Engineering, Queen’s University Belfast, Belfast, BT9 5AG, United Kingdom; d Sustainable Agri-food Sciences Division, Agri-Food and Biosciences Institute, Belfast, United Kingdom; e Department of Natural Resource Sciences, McGill University, Montréal, Canada

**Keywords:** Agriculture, Northern Ireland, Participation, Scenario analysis, Social learning, Substance Flow Analysis, Transformations

## Abstract

Food systems worldwide are vulnerable to Phosphorus (P) supply disruptions and price fluctuations. Current P use is also highly inefficient, generating large surpluses and pollution. Global food security and aquatic ecosystems are in jeopardy if transformative action is not taken. This paper pivots from earlier (predominantly conceptual) work to develop and analyse a P transdisciplinary scenario process, assessing stakeholders potential for transformative thinking in P use in the food system. Northern Ireland, a highly livestock-intensive system, was used as case study for illustrating such process. The stakeholder engagement takes a normative stance in that it sets the explicit premise that the food system needs to be transformed and asks stakeholders to engage in a dialogue on how that transformation can be achieved. A Substance Flow Analysis of P flows and stocks was employed to construct visions for alternative futures and stimulate stakeholder discussions on system responses. These were analysed for their transformative potential using a triple-loop social learning framework. For the most part, stakeholder responses remained transitional or incremental, rather than being fundamentally transformative. The process did unveil some deeper levers that could be acted upon to move the system further along the spectrum of transformational change (e.g. changes in food markets, creation of new P markets, destocking, new types of land production and radical land use changes), providing clues of what an aspirational system could look like. Replicated and adapted elsewhere, this process can serve as diagnostics of current stakeholders thinking and potential, as well as for the identification of those deeper levers, opening up avenues to work upon for global scale transformation.

## Highlights

• Transdisciplinary process on potential for transformative thinking in P use.
• Substance Flow Analysis of visions for alternative futures and system responses.
• Analysed for their transformative potential using triple-loop social learning.
• Stakeholder responses remained transitional or incremental, not transformative.
• But some deeper levers that could be acted upon to transform the food system.

## 1 Introduction

Phosphorus (P) has no substitute in food production and all food systems are now dependent on fertilisers derived, in large part, from a finite supply of phosphate rock. This supply is controlled by a handful of countries, with Morocco alone controlling over 70% (Cordell and Neset, 2014, USGS, 2021). This creates major uncertainties over continued access (Blackwell et al., 2019) and makes national and regional agri-food systems, and global food security more broadly, potentially vulnerable to supply disruptions and price fluctuations (Cordell and Neset, 2014). At the same time, current P use is highly inefficient (e.g. an analysis of P stores and flows across Europe in 2005 showed a P system efficiency of only 38% (Dijk et al., 2016; Withers et al., 2020). This creates wasteful system that extracts P for food production and generates large surpluses in agricultural soils, causing pollution of water resources and compromising aquatic biodiversity worldwide (Elser and Bennett, 2011), resulting in decades long transgression of planetary boundaries (Carpenter and Bennett, 2011). Global food security and healthy freshwater and coastal ecosystems are, consequently, jeopardised if no transformative actions to make P use more efficient are taken across the food, agriculture and waste sectors (Brownlie et al., 2021).

This dispersal of P in the system is the result of a ‘chaotic’ governance in terms of its distribution, usage, loss and accumulation (Withers et al., 2020). At present, no global framework for P governance exists and its management is largely ignored in most international policy discussions (Brownlie et al., 2021). In countries where regulation does exist, it is dated and fails to address sufficiently the wider aspects of sustainable use to support global food security (Reitzel et al., 2019). Consequently, attempts to improve P-use efficiency and sustainability within food systems have so far been reductionist, with emphasis on removal of P in wastewater effluent discharge and agronomic solutions (such as recycling biosolids to agricultural land or reducing P farm inputs), ignoring P inefficiencies that occur at other stages in the food supply chain (Withers et al., 2020). These wider food chain inefficiencies are the consequence of societal functions involving diverse sectors and a wide range of stakeholders, whose roles and responsibilities in the P challenge are ambiguous (Lyon et al., 2020).

Transformative system changes that transcend the limitations of reductionist solutions are needed (Withers et al., 2020). Transdisciplinary approaches where a diversity of knowledges –including non-academic- and values are brought together (Fazey et al., 2018, Reed, 2008), have been widely proposed as part of the strategy to deal with this type of ‘wicked’ problem (Lyon et al., 2020). These approaches are based on the idea that technocratic siloed approaches are unlikely to provide adequate responses to the inherent complexity of such socio-ecological challenges (Duckett et al., 2016). Transdisciplinary approaches have the potential, through individual and group interactions, of fostering social learning, contributing to overcome the development of new institutions, norms and multi-level network interactions required for transformation (Pahl-Wostl et al., 2009). In the global P challenge, a collective deeper understanding of P dynamics at global to regional scales, as well as the clarification and empowerment of stakeholders over the role they can play in those dynamics, are needed to explore transition strategies towards such transformative change (Cordell et al., 2015, Lyon et al., 2020, Withers et al., 2020a).

Despite the emphasis that the literature has put on the need for stakeholders and actors to engage with the P issue (Scholz et al., 2013, Scholz et al., 2014, Withers et al., 2020a), there are very few empirical case studies that bring them together in participatory processes. Those that exist have focused on building conceptual models of change (e.g. in North America (Jacobs et al., 2017) and Ireland (Macintosh et al., 2019)) arising out of discussions with a range of government, non-government, research and industrial stakeholders. This paper pivots from this earlier predominantly conceptual work, to develop and analyse a P transdisciplinary scenario process, assessing stakeholders potential for transformative thinking in refocusing P use in the food system, illustrated through an example in Northern Ireland. We use Substance Flow Analysis (SFA) of current and alternative P stocks and flows to illustrate alternative visions for the future of P in the region’s food system and, through participatory scenario analysis (Oteros-Rozas et al., 2015), to stimulate stakeholder discussions on system responses and strategies towards more sustainable and resilient P use. We analyse these discussions in terms of social learning (Pahl-Wostl, 2009) and use this to reflect on the region’s position on a transformational pathway. While the specificities of the discussions may be of relevance to other livestock-dominated systems, the process itself is expected to be useful worldwide.

## 2 Methodology

Situated within the constructivist research paradigm (Guba et al., 2018),^1^ qualitative participatory scenario analysis is increasingly being applied in diverse environmental research contexts (Oteros-Rozas et al., 2015). It aims at articulating alternative descriptions of the future that can be used to explore possible consequences of decisions in a changing and uncertain world (Mietzner and Reger, 2004). Scenarios are not predictions, but plausible, internally coherent descriptions of future states (Waylen et al., 2015). It allows stakeholders to engage in a collaborative process to investigate alternative futures, usually in a solutions-oriented way. As explained by Oteros-Rozas et al. (2015), the rationale for stakeholder engagement in scenario analysis follows normative arguments related to broader participation discourses in environmental management to empower stakeholders, to stimulate innovation, to mitigate conflicts and encourage social learning, and to integrate different types of knowledge (e.g., scientific, local), perceptions, expectations, and aspirations. Involving diverse stakeholders with influence and interest in the social-ecological system can foster social learning and collective action to achieve desired goals (Oteros-Rozas et al., 2015). Participatory scenario analysis is therefore particularly relevant for this research’s aspiration to trigger a conversation on refocusing the use of P in the food system, since it can elicit how stakeholders might respond to future challenges and help identify adaptation pathways (Brown et al., 2016). In our case, the stakeholder engagement takes a normative stance in that it sets the explicit premise that the food system needs to be transformed and asks stakeholders to engage in a dialogue with us (the researchers) on how that transformation can be achieved.

A SFA of current P in Northern Ireland was constructed with input from a diverse set of stakeholders representing the agri-food, environment and waste sectors (Rothwell et al., 2020). SFA is a modelling approach used to visualise and analyse the imports, flows, stocks, losses and exports of a material from a defined system using a mass balance and mass conservation approach (Brunner and Rechberger, 2017). A range of SFA of alternative futures were developed by the researchers, representing diverse scenarios of P use. These were then employed in a workshop with stakeholders to discuss visions for alternative futures in which P was used differently across the various relevant food, agriculture and waste sectors. Used in this way, the SFA represents a novelty over previous stakeholder processes in the P literature (Carraresi et al., 2018, Cordell et al., 2021, Jacobs et al., 2017, Macintosh et al., 2019).

Stakeholders’ discussions were analysed in terms of how far they were able to shift to a transformational position to achieve more sustainable P use, using Pahl-Wostl (2009)’s ‘triple loop’ framework on social learning. Like Brown et al. (2016), we used the learning loops as a diagnostic tool for referencing the transformational potential of stakeholders’ responses, stimulated by the SFA participatory scenario discussion. The ‘single-loop’ represents a consolidative process that is primarily structured around reactive or incremental actions without major changes in mental models. The ‘double-loop’ involves actors changing their reference frame and guiding assumptions to identify new ways to achieve strategies or goals. This requires challenging and possibly changing existing rules. The ‘triple-loop’ takes actors beyond pre-existing structures by challenging existing decision paradigms and the context which frame the decision-making processes, including underlying principles and norms (Table 1).

**Table 1:** Key characteristics of single-, double- and triple-loop learning from Pahl-Wostl (2009) and modified by Brown et al. (2016).

	Single loop: incremental improvements of established routines	Double loop: reframing of issues and challenging of assumptions	Triple loop: transformation of structures and regimes
*Institutions*	Existing established institutions	Reinterpretation to encourage innovation beyond established groups	Institutional change or new institutions to enable new paradigms
*Norms*	Established norms	Norms questioned	Actions based upon new norms
*Actor networks*	Same actor networks	Roles and identities questioned; new networks considered	Change in networks, roles and power relations
*Multi-level interactions*	Established vertical patterns	Increased informal knowledge exchange between levels	Polycentric structures; formalised participation and knowledge exchange at different levels
*Governance*	No change in dominant mode	New governance types become visible (e.g. market based instruments)	New and diverse types of adaptive governance implemented
*Uncertainty*	Risk-averse with limited adaptation and aim to “reduce uncertainty”	Uncertainty used to identify different perspectives and frames	Uncertainty emphasises different perspective and adaptive approaches

### 2.1 Case study: Northern Ireland’s Phosphorus challenge

Northern Ireland represents a prime case for investigation in such context. Similar to many regions and countries across the globe, the emphasis on P management within Northern Ireland, has focused on improving agricultural efficiency and reducing losses to water within a livestock-dominated region (Barry and Foy, 2016, Kleinman et al., 2015). This has largely been due to the important role that livestock agriculture plays culturally and economically, with the value of food and drink sold to markets outside of Northern Ireland over £ 3.6 million, predominantly from livestock-derived (DAERA, 2019).

While there has been significant improvement in water quality (Barry and Foy, 2016) since the 90’s, currently only 31.3% of river waterbodies and 24% of lakes are achieving the target of ‘Good Status’ required under the European Water Framework Directive, with P inputs from agriculture highlighted as the single biggest cause of failures to meet the targets (DAERA, 2018; Rothwell et al., 2020).^2^

While P use efficiency has increased from 28%, prior to the implementation of the Nitrates Directive in 2003, to 42% in 2017, the annual agricultural P surplus has only fallen below 10 kg/ha in three out of the past fifteen years (up to 2017) (Rothwell et al., 2020). In 2017 the annual P surplus was 12.3 kg P/ha, up from a low value of 8.7 kg P/ha in 2008, with a target of 5 kg P/ha being the government’s stated objective (Rothwell et al., 2020). A recent government soil sampling scheme has demonstrated the impact this agricultural surplus has had on soil P levels with 38% of soils having P concentrations above the optimum for grassland (Higgins et al., 2020). The agri-food industry has struggled to keep the national balance below 10 kg P/ha, largely due to an increasing reliance on imported concentrate feeds in the livestock sector and continued use of chemical fertiliser despite the availability of surplus nutrients in manures. In addition to the risk posed to water quality from these high P soils (Cassidy et al., 2019, Cassidy et al., 2017), the spatial and temporal distribution of manure P is a critical issue for the future sustainability of Northern Ireland agriculture. The combination of localised intensive livestock production, limited availability of arable land, cost of transporting slurry, limited infrastructure for slurry processing and 57% of soil classed as high risk of runoff (Cruickshank, 1997) pose significant challenges for farmers in terms of balancing agronomic and environmental objectives.

### 2.2 Stakeholder recruitment and participation

Stakeholders were recruited following the approach proposed in Lyon et al. (2020) for system transformation and that pays special attention to reflecting system complexity. As such our stakeholder recruitment is driven by looking at the positioning of key stakeholders (Herepath, 2014), in this case those representing organisations that define the P use and policy environment in Northern Ireland.

A preliminary list of sectors was prepared collectively by the multi-disciplinary research team. One of the partners participating in this research, the Agri-Food and Biosciences Institute (AFBI), who has a longstanding relationship with most key stakeholders of the agri-food scene in Northern Ireland, carried out the actual recruitment (i.e. send the invitations, follow-up with contacts and snowball to further participants). This longstanding relationship allowed us to exhaust the list of potential participants during our recruitment. The aim was to engage the range of institutional stakeholders affected by and likely to be able to exert influence over P sustainability, including the main farmers’ representative organisation^3^ wastewater companies, government regulatory agencies, policymakers, fertiliser companies, and scientists (Lyon et al., 2020).

Stakeholder engagement began with those who were able to provide direct input (data, information or feedback) for the creation of the current SFA. A cross section of all relevant stakeholders were then invited to participate in the workshop, which included group discussions and an individual reflection form (Table 2).^4^

**Table 2:** Participating stakeholders.

Sector	Area	Name of organisation	Participation in the research*
Waste management	Renewable energy / Waste recycling	Granville Eco Park	a
	Waste disposal	ISL Waste Management	a
	Organic waste	Natural World Products	a
	Anaerobic digestion / Energy production	Stream Bioenergy	a, b, c
	Environment / Resource efficiency	WRAP	a, b, c
	Waste processing	AgriAD	b
Government Agency	Data management –Resource efficiency	Department Of Agriculture, Environment And Rural Affairs	a
	Waste Recycling	Department of Agriculture, Environment and Rural Affairs	a
	Slaughter waste disposal	Department of Agriculture, Environment and Rural Affairs	a
	Economics and evaluation	Department of Agriculture, Environment and Rural Affairs	a
	Environmental and farming policy	Department of Agriculture, Environment and Rural Affairs	a, b, c
	Science and policy	Department of Agriculture, Environment and Rural Affairs	b
	Regulation and natural resources policy	Department of Agriculture, Environment and Rural Affairs	b, c
	Industrial waste and consents	Northern Ireland Environment Agency	b, c
	Environment / Water quality	Northern Ireland Environment Agency	b, c
	Environment/ Water Quality	Northern Ireland Environment Agency	b, c
	Farming and environment	Northern Ireland Environment Agency	b, c
	Evidence and monitoring	Northern Ireland Environment Agency	b
	Emissions and land management	Department of Agriculture, Environment and Rural Affairs	b
	Industry pollution regulation	Northern Ireland Environment Agency	b
	Farm regulations	Department of Agriculture, Environment and Rural Affairs	b
Animal feed	Agri-Technology and Sustainable farming	Devenish Nutrition	a, b
Food	Poultry	Moy Park	b, c
Research	Catchment Modelling	AFBI	b, c
	Nutrient management	AFBI	b
	Nutrient management	AFBI	b
	Renewable energy/technology	Queen’s University Belfast	b
	Pig and Poultry	AFBI	b
	Phosphorus recycling technology	Queen’s University Belfast	b
	Nutrient management/Anaerobic digestion	AFBI	b
	Nutrient management / Renewable energy	AFBI	b
NGO	Environment/conservation	Ulster Wildlife trust	
Water utility	Waste management	Northern Ireland Water	b, c
	Water management	Northern Ireland Water	b, c
Farming	Farmer advocacy group	Ulster Farmers Union	b, c

* (a): feeding into the current SFA (providing data, feedback or access to information); (b) participating in the scenario workshop; (c) participating in the individual reflection form.

### 2.3 The current Substance Flow Analysis

**Fig. 1:**
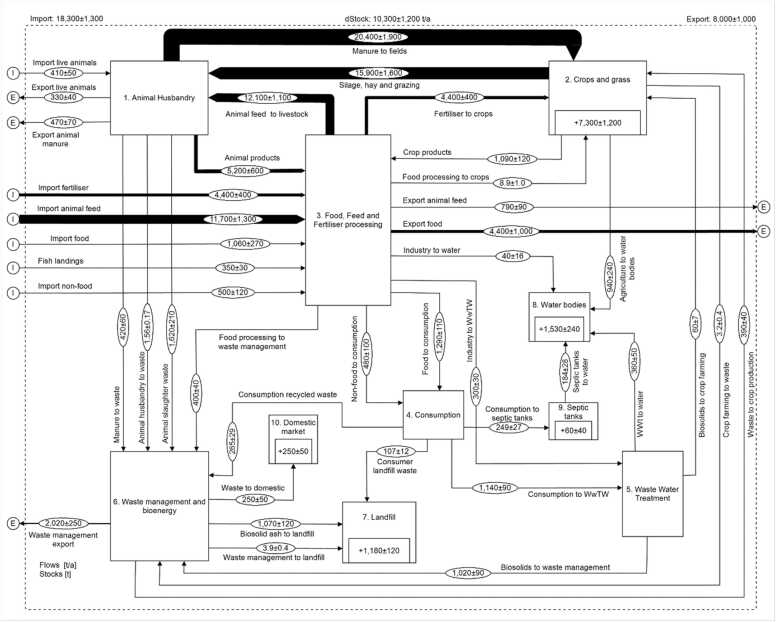
*Phosphorus Substance Flow Analysis for the current Northern Ireland food system.* Note: The SFA shows It shows a total (including non-food P) of 18,300 t of P imports and 8000 t P exports, leading to a surplus of over 10,000 t of P. Of this, 71% accumulates in the soil, while 15% is lost to water, with the rest accumulating in landfill, septic tanks or domestic markets as non-food products (e.g. compost). The SFA shows a food system P efficiency (i.e. system product/system input) of 38%, with 17,900 t of inputs (coming from imports of animal feed, fertilizer, food and live animals and fish landings) and 6810 t of outputs (to food exports, Northern Ireland food consumption and exports of animal feed and live animals). Highlights of sector by sector flows and efficiency as presented to workshop participants are presented in the Supplementary Materials and more details on the analysis can be found in Rothwell et al. (2020). Full details of the SFA and its build up available in Rothwell et al. (2020).

SFA is used here as a tool to visualize system parameters, synergies and feedback loops across the wider food system (i.e. beyond individual sectors and, importantly, transcending the reductionist agronomic focus), and was employed to simulate the current P flows in Northern Ireland (Brunner, 2010). Data used included national statistics, industry published annual reports or previously published scientific data and via stakeholder input in a consultative participation process (Mobjörk, 2010). This consultative participation also served to ‘ground-truth’ (Martin-Ortega et al., 2015) or validate some of the estimates and flows, leading to the refinement of what we refer to as the ‘current SFA’ (Fig. 1).

### 2.4 Workshop design and implementation

A one-day workshop, advertised as “Phosphorus Substance Flow analysis of Northern Ireland’s food system”, was organized in February 2020. Participants were informed that the overall aim of the research was to refocus P use in the UK food system, with the broader aspiration to trigger a conversation on the transformation of the UK’s food system for a more P resilient future. They were informed that regional food systems (like Northern Ireland’s) are reliant on P but use it very inefficiently and that P losses to water are causing extensive eutrophication leading to loss in biodiversity and risks to human health and well-being. They were told that over-reliance on mined P has increased food system’s vulnerability to shocks and that climate change is likely to exacerbate the P problem. Examples of the general strategies of stewardship required for sustainable P use against a backdrop of accelerating nutrient use to meet increasing global food demand were outlined. Questions such as how do we change systems to improve P efficiency, sustainability and resilience and how do we enact the changes required for future food and water security, were used as introductory framings for the day. The introduction included a brief description of the background of P management in the region. Participants were then presented with key findings of the current SFA as summarized in Section 2.3 and in the Supplementary Materials.

The participatory scenario exercise took place next. Setting up two goals: reducing P exports to water and sustaining food system integrity (i.e. profitable business, sustained livelihoods and food security), the exercise allowed discussion of alternative scenarios that represented variations of the current situation. Using the current SFA as a baseline, five new P SFAs, where significant parts of the food system were changed to alter their P flows, were presented. Participants were informed that the scenarios were not about likelihood, but about possibility. Differences of the SFA scenarios where highlighted in terms of: *Surplus*, the level of excess P above that required by the food system; *Predicted river Soluble Reactive Phosphorus (SRP)* based on estimated of the relationship between the national P surplus and SRP in 96 rivers across NI; *P import*, this is a measure of the total P being imported into the food system and is reflective of the economic cost of P to the system; and *Food system efficiency,* a measure of how efficient the food system is at converting P imports into food and usable commodities (Table 3).

**Table 3:** Scenario labels and key metrics presented to stakeholders.

Scenario label	System change	Key metrics		% change from current
Current	No change	Surplus (kg/ha)	8.5	0
		Predicted river SRP (ug/l)	58	0
		P import (t/yr)	18,337	0
		Food system efficiency (%)	38	0
Scenario 1: Manure export	35% of manure P is ‘processed’ via waste management and exported.	Surplus (kg/ha)	0.16	-98
		Predicted river SRP (ug/l)	31	-46
		P import (t/yr)	18,334	0
		Food system efficiency (%)	38	0
Scenario 2: Legacy P	Fertiliser P application is reduced by 95%.	Surplus (kg/ha)	5.7	-33
	Manure P application is reduced by 41% Crops/grass draw down existing soil P at a rate of 5.5 kg/ha/Excess manure P is exported.	Predicted river SRP (ug/l)	47	-19
		P import (t/yr)	14,403	-22
		Food system efficiency (%)	41	+ 3
Scenario 3: Diet change	Changing global dietary habits leads to 25% reduction in consumer demand for animal food products. Feed and fertiliser P, grass production P, livestock produce P and food export P all reduced by 25%.	Surplus (kg/ha)	5.7	-33
		Predicted river SRP (ug/l)	47	-19
		P import (t/yr)	14,403	-22
		Food system efficiency (%)	41	+ 3
Scenario 4: Target 1.5	Fertiliser P use reduced by 75%. Animal feed P concentration reduced to 0.35% with no impact on productivity. Manure P input reduced by 20% due to lower feed P inputs.	Surplus (kg/ha)	1.6	-81
		Predicted river SRP (ug/l)	35	-40
		P import (t/yr)	12,269	-33
		Food system efficiency (%)	58	+ 20
Scenario 5: Balanced System	No P fertiliser is used. All post farm food system waste P is recovered and recycled. Only manure needed to meet crop P demand is used. 30% of manure P is exported.	Surplus (kg/ha)	0.22	-97
		Predicted river SRP (ug/l)	31	-46
		P import (t/yr)	13,922	-24
		Food system efficiency (%)	52	+ 14

The scenarios were given easy to retain names: 1 – *Manure export*, where technologies enable the processing of livestock manure that facilitates economically viable export outside of Northern Ireland. 2 – *Legacy P*, where manure and fertiliser P inputs are reduced to a level that mean crops rely on the existing reserves on P that have accumulated in the soil due to historic over-application. 3 - *Diet change*, where current trends for increased veganism mean demand for livestock food products reduce by 25%, consequently, the livestock population of Northern Ireland and associated feed P and manure P flows are reduced by a quarter. 4 – *Target 1.5*, in which the agricultural P surplus in Northern Ireland is reduced to 1.5 kg/ha by altering fertiliser, animal feed and manure P flows, analysis suggests this would reduce river SRP to a target level of 35 ug/l. 5 – *Balanced system*, in which all P soil inputs are reduced to levels that meet crop demand, i.e. the surplus is reduced to zero, making it closest to P circularity in the food system.

Participants were asked to vote for the two scenarios that they would most prefer to discuss.^5^ They overwhelmingly voted for the *Legacy P* (scenario 2) and the *Target 1.5* (scenario 4). Scenario 5 (*Balanced system*) was included in the discussions by the research team because it included aspects potentially relevant to all sectors. Votes were individual and participants could keep their vote secret.

In break-out groups, participants were asked to discuss organisation/sector impacts and responses to each of the scenarios, using a carousel format. The frame used was: “imagine you wake up tomorrow and this [scenario] is the new situation”. Participants were stimulated to develop ‘blue-sky’ thinking, taking the scenarios as a ‘barrier free’ situation, i.e. with no constraints to responses, such as budget or legislation constraints. A second discussion session focused on what the main barriers would be (e.g. financial, cultural, regulatory or technological) to achieve the goals (i.e. reduce P exports to water and sustain food system integrity) under their new scenario, and what levers could be acted upon to lift those barriers.

After a plenary session in which participants were given the opportunity to provide further feedback or make any additional contribution, they were asked to individually fill in a form with their personal reflections on the day.^6^ Twenty participants filled in the reflections form.

### 2.5 Data analysis

This research uses qualitative data analysis of the materials recorded at the workshop and the individual reflection forms. Break-out and plenary discussions were recorded during the workshop by a note-taker in flipcharts. These notes were visible to all participants and at break-out groups the facilitator asked for validation of the notes by the members of the group, as well as for clarification or further development of any unclear issues. Recorded in this way, the notes were then transcribed and compiled in a dataset of 137 statements or nodes (i.e. each argument of the discussion as recorded by the note-taker correspond to a node). Structured coding (DeCuir-Gunby et al., 2011) was used to analyse the data, with the support of the Nvivo12 software. Statements were first coded for the six domains of the triple-loop framework (i.e. institutions, norms, actors networks, multi-level interactions, governance and uncertainty) and then secondly, to one of the first, second and third loops (as per Table 1). Our structured coding was initially carried out independently by two members of the research team. This led to disagreements over 33 nodes (24% of the total). A third researcher independently coded the nodes over which there had been disagreement, which were then reduced to five after discussion among the three researchers. The coding was then shared with the rest of the interdisciplinary research team, until final consensus was achieved.^7^

After removing duplicates, a total of 121 statements were retained. Inductive coding was used to identify further relevant themes in the data from the workshop and the reflective forms, providing context to the multi-loop learning analysis.

## 3 Results

### 3.1 Workshop discussions

Results from the workshop discussions are presented next through a summary of the discussion points in terms of the impacts and responses, barriers and levers, followed by the results of the multi-loop analysis in relation to the six domains in Pahl-Wostl (2009)’s framework. There is not always a clear cut distinction between some of the domains in Pahl-Wostl’s framework (e.g. between institutions and governance, or norms and governance). The three-way independent coding applied in our analysis, tried to harmonize this as much as possible, but it is to be acknowledged that different analysists might have classified some arguments differently across these domains. In any case, however, the emphasis of our analysis is on the three loops and their indication of stakeholders’ coping, adaptive or transformative thinking, the domains are merely used as means to present the information more easily. The classification of the stakeholder arguments in each of the loops is highly context dependent, i.e. what might be seen as little change over the business as usual in one context, might be quite radical in Northern Ireland’s context and vice versa.

Next we present the results for each of the scenarios, with the details of the statements and their classification in the three loops presented in the Supplementary Materials.

#### 3.1.1 Scenario 2 - Legacy P

The foundation of this scenario is rooted in harnessing existing legacy P in soil. If the agricultural industry began utilising the P already in the soil for plant growth, this would in theory, reduce the dependence on external P input to the system (LeNoë et al., 2020). At present 38% of the soils in Northern Ireland are estimated to be above the agronomic optimum for crop growth as measured by a soil P test (Higgins et al., 2020), and there are large stores of soil P not currently measured by soil P tests which are additionally potentially available over time (Rowe et al., 2016). The impacts of this scenario mentioned by workshop participants included potential future soil P test deficit at farm and regional scales, and a potential knock-on effect on productivity. In this scenario, all excess manure is to be exported from the system which, as discussed by participants, would require increased processing and steps to support energy production. The diversion of manure flows to anaerobic digestion (AD) and energy production was agreed in the group as a main response, along with developments/re-orientation of technology to support this. Clear positive impacts included the results on water quality and a increase in jobs in the energy sector. Looking at the entire agri-food system, participants discussed a change in future food markets, as this scenario was seen to be coupled with a switch of products from agriculture to new markets aligned with different farming methods.

The main points of discussion regarding barriers related to the practical aspects of lack of facilities to deal with manure processing and a lack of market for any new derived products. Participants felt that the unknowns in this scenario are plentiful, ranging from the impact of climate change, to how the specific player attitudes could or could not change. Another dominant unknown was related to time frames i.e. how long would this system have to exist to balance the P flows, and how long actual drawdown of soil P would last as this “*is seen to reduce over time*”. Discussions on levers emphasised the need for more research and scientific evidence to support this change in the agri-system.

#### 3.1.2 Scenario 4 – Target 1.5 kg/ha

This scenario includes system changes that limit the P surplus to 1.5 kg/ha, the estimated surplus required to achieve a water quality target of 35 ug/l. These changes were a reduction of fertiliser P use by 75%, a reduction in animal feed P concentration from the current level of 0.46–0.35% (with no impact on productivity) and a manure P input reduction of 20%. This triggered a conversation on the economic impacts of this scenario, but there was no consensus on how farmers would be affected. On one hand, it was argued that it could be a benefit due to lower fertiliser costs, but counter to this, feed costs might increase due to sourcing of low P feeds, and farming practices (such as livestock intensity) may have to change which might reduce profit. While some stakeholders considered potential outcomes were positive and optimistic, others anticipated potential negative (economic) impacts on the feed industry in the form of additional cost of production. One industry that dominated discussion was dairy farming and a reduction in P concentration in the feed was considered to negatively impact dairy farming, either as a drop in production, or an increase in feed costs.

On how the system would respond to these changes, stakeholders highlighted the need for an increase in manure processing facilities and more innovative cropping practices to better harness P inputs, for example alternative industrial crops (hemp/energy crops). A new set of products from the fertiliser sector to suit changes in demand was also mentioned as a response.

Regarding barriers, a predominant argument referred to lack of knowledge relating to willingness to change and market demand. More practical barriers included those associated with manure transport and processing for example biosecurity and the spread of disease like tuberculosis. A new barrier mentioned under this scenario was how the culture of farming might restrict how far management practice could change in Northern Ireland. Coupled with these barriers were levers such as the need for more research, innovation, and knowledge transfer. The provision of incentives to develop and enforce effective regulatory systems to support change was also viewed as an important lever.

In general, scenario 4 was regarded as the most achievable in terms of knowledge, technology and stakeholders’ ability and willingness to adapt. Positive attitudes though were associated with the feeling that it is the *“least painful”* to achieve because no major restructuring of the food system per se would be required. Participants mentioned that the *“Idea is very good because there is a need to reduce the surplus”* but some also felt that the scenario would “*not be enough to solve the problem*” in Northern Ireland. It was mentioned that targets like this have been aimed for in the past, suggesting that it had not been enough and that further actions are needed.

#### 3.1.3 Scenario 5 - Balanced system

In this scenario, P inputs to the system exactly meet crop P demand, aiming for a P surplus of zero. Important changes in the system include fertiliser use reducing to zero, and P from manure being utilised at a level which matches crop P demand, thereby creating a circular P economy, with all post farm food system P being recovered and recycled. However, due to the current P surplus within the agricultural system, wastewater biosolids are not returned to land, and therefore any recovered P would have to be stored or exported.

Discussions on the impacts of this scenario centred predominantly on the waste-water treatment sector. Frequent mention of the terms “*redesign*” and *“re-engineer”* highlighted the need to recover higher volumes of P from the wastewater treatment process steps. An attitude *“P recovery is feasible”* applied to both waste-water processing and manure management. Scenario 5 was also felt to challenge the fertiliser industry and potentially push this sector towards a change in formulations, products and markets. A dominant view was the concept of ‘change’. Across all the farming, fertiliser, feed and wastewater sectors the consensus was that more fundamental change would be needed to deal with the impacts and the characteristics of this scenario, including a change of land use and in methods dealing with food and crop waste.

The barriers section of the discussion was dominated by comments involving farmers and their interaction with consumers. Farming sector barriers included tension between livestock practices and consumer preferences, practical restrictions on recovering P and lack of sufficient subsidies to move toward greater integration of livestock and arable farming systems. In the lever discussions, although interventions were mentioned to facilitate changes in the farming community, it was noted that this might favour some farmers over others (in the form of payments). This social aspect was carried through to the multiple unknowns under this scenario, for example “*are the interventions socially acceptable for the farmer outside the target area?*” (i.e. if subsidies are given in targeted areas, would the farmers outside these areas be/feel disfavoured?).

There seemed to be a general feeling that a “*new framework*” was needed to allow the required changes to happen and maintain public and political confidence in the food system, and that this framework would need to be regulated. While it was unclear what a new framework would entail, it does indicate a sense of structural need for change.

A number of arguments came across all scenarios. Concerns were voiced of how farmers might perceive any new prescription for change that may emerge from the alternative scenarios. Reduction in stocking density also appeared in conversations across the various stakeholders, clearly emerging as a controversial topic, with some stakeholders advocating for the need to t “*break up the de-stocking ‘taboo’*”, with others claiming that farmers would never accept it.

Biosecurity in relation to the transport of P from farm-to-farm was also seen as a barrier across all scenarios, although increasing the solid proportion of manure was seen as a mechanism to address it. Other recurrent aspects of the discussion included a series of questions on how to monitor the changes i.e. “*how do we detect increased runoff from the land? How we assess if the changes are happening? Do the water pollution levels react from changes in application volume?”.* These unknowns are coupled with an emphasised lever of identifying which metric is the best to use to monitor the impact of change and communicate those back to stakeholders. Further, stakeholders repeatedly highlighted that new technology may not always be needed, instead existing technology could be harnessed and adapted to meet the needs of the new scenarios.

### 3.2 Reflections forms

Generally, stakeholders’ reflections suggest that their views about the future of Northern Ireland’s food system did not change substantially after the workshop, but were mainly reaffirmed. For instance, some reported that the workshop consolidated their view that the food system was not well balanced and there is scope to improve. Others noted that they thought P needs to be exported out of Northern Ireland and that this belief was strengthened as a result of the discussion of all scenarios. In contrast, however, a few people reported that the SFA models caused them to reconsider destocking and agri-environment schemes in particular, and broadened their understanding of the challenges associated with P management.

A few stakeholders noted that they gained greater insight into technicalities and barriers in some sectors. As a result, some stakeholders indicated that *“we don’t necessarily need completely novel approaches”,* though efforts are required in the following areas: collaborative research across sectors and different stakeholders, with a focus on effective routes to engagement and what kind of evidence or information is needed to make it meaningful; increasing investment in the provision of good advice on P stewardship; and ensuring that different actors, policy makers and members of the industry engage, appreciate the barriers to change and look at how to overcome and develop solutions.

SFA made clear that P use and impacts has a potential interaction with other issues (e.g., climate change, ammonia). This was also a recurrent argument in the workshop across all scenarios, i.e. the *“need to look at P not in isolation”* – which may differ in priority for different sectors and stakeholders. Thus it was perceived that a collective approach, involving all key stakeholders, would be required to achieve a “*sustainable circular economy*”.

## 4 Discussion

The stakeholder engagement process carried out in this research, clearly showed that there is a general understanding amongst stakeholders that P use in Northern Ireland is indeed inefficient and that there is scope to improve P efficiency so as to reduce environmental damage and increase the system’s resilience. While P have been in policy discussion in Northern Ireland for a couple of decades now (Foy et al., 2002, Gibson et al., 2001, McGuckin et al., 1999), the emphasis has been on improving agronomic use efficiency and reducing losses to water, in line with the reductionist approaches that have been criticised by the literature (Withers et al., 2020). Vulnerability to P scarcity and its potential impact on feed and fertiliser import have only recently entered the debate in the region (Macintosh et al., 2019). In this context, the SFA proved to be a very useful tool for enlarging the scope of the discussion on the broader role of P in developing sustainable food systems. The scenarios acted as effective boundary objects allowing for discussion about alternative futures for P in the system, enabling conversations to occur in a more systemic and holistic way, as had been intended (Oteros-Rozas et al., 2015). When linking the supply–demand chain view of P with the SFA, the key actors in the P cycle became evident, as well as the hotspots and flows of P vulnerability, serving as a very effective tool to understand the role of P in the system (Scholz et al., 2014).

This conversation led to an array of collective reflections on potential stakeholder and sector responses. The multi-loop social learning analysis showed that, to a large extent, these remained within the remit of coping and adaptation strategies (first and second loops), rather than achieving particularly transformative thinking (third loop). Responses placed the focus predominately into better managing the parameters and feedbacks of the existing food system (Withers et al., 2020), making use of what can be referred to as shallow levers, which are likely to bring little change to the overall functioning of the system. These are reflected in our results (Tables 4–6 in the Supplementary Materials) for example, in relation to better soil management to make better use of P in the soil (11), making soil testing compulsory across the whole of Northern Ireland (5), reducing dependence on chemical fertilisers and feed (47, 48), and optimising P in the grass (87). Related themes include more widespread monitoring and testing (84, 85), increasing communication, knowledge transfer and farmers’ advice, training and options (17, 19, 20, 45, 58, 64). Internal synergies towards P recycling related to abundant calls to changes in the perception of manure and slurry as resources (8, 49, 51, 61, 62, 90, 100) and calls for circular bio-economy (9). Relevant here is the importance given to the role of anaerobic digestion (AD) and the linkage with the energy sector, with recurrent calls for the diversion of manure flows to AD and energy production (1, 12, 65, 66, 94), further engaging the industry (7) and moving beyond just the environmental and farming sectors. Policy responses ranged from enforcement, compliance and incentives (22, 23, 37) - including paying farmers for the delivery of (P) public goods (3) - more focused funding and targeted and integrated interventions (24, 25, 86).

The fact that Scenario 4 was highlighted as the most achievable is also aligned with this idea that stakeholders did not envision a particularly transformative process when prompted. This target could be achieved by a combination of reducing P fertiliser use and the P content of animal feed concentrates. While it would also require manure P export from Northern Ireland, in a way, this scenario is the one that would require the least transformative change across the whole system (or as mentioned explicitly in the workshop by participants: *“no major re-structuring required”*(38)). Stakeholders themselves, however, acknowledged that this would probably “*not solve the problem*”. This perception may be due to the legacy soil P issue in Northern Ireland that may require a negative P balance to reduce soil P concentration to levels that are sustainable in the longer-term. In addition, there is uncertainty as to whether a P balance of 1.5 kg ha^-1^is agronomically sustainable for meeting the higher production goals required of livestock farming in Northern Ireland (Agri-Food Strategy Board, 2015).

A repeatedly mentioned barrier in the discussion were the unknowns associated with the scenarios and with the future of farming support in general, which has also been found in other stakeholder based P research (Carraresi et al., 2018). This appeared to impede the ability of the stakeholders to make a case for the plausibility of the system changes. Indeed, uncertainty was present throughout responses across the scenarios, but mostly framed in terms of a limitation to adaptation and in relation to the aim of reducing it. Mentioned uncertainties range from specific technical knowledge, e.g. regarding legacy soil P and its use (29, 34), economic implications (31, 33), to broad ranging, generic uncertainties around climate change, infrastructure needs, attitudes, etc. (27, 28, 30, 32, 77, 78). No new approaches to manage uncertainty and risk were mentioned as mechanisms to change structural constraints (third loop), staying within the realm of relying on science to fill the knowledge gaps and reduce the uncertainty (Pahl-Wostl, 2009).

System wide transformative change requires “deeper” leverage points by which the goals of a system, its intent, and rules are reconsidered (Dorninger et al., 2020). Such transformative changes require for those involved to both think and act differently based on a different set of assumptions, concepts and norms (Abson et al., 2017, Pahl-Wostl et al., 2011). Such processes are notoriously difficult, since pre-existing ways of thinking, working and governing tend to be ‘sticky’ (Walyen et al., 2015). For example, despite strong evidence over the past 20 years to support the need for regular soil sampling to inform sustainable nutrient management, it is only recently that this has become a key policy objective (Higgins et al., 2020) and it has still not been widely adopted on farms across Northern Ireland. Further, these processes are likely to bring uncomfortable discussions around current environmental governance (Pahl-Wostl et al., 2011). This was the case in relation to destocking in our case study. Although strongly advocated by the environmental lobby, destocking has not yet been seriously considered in Northern Ireland in relation to achieving the sustainable management of P, as it has, for example, in other very heavily livestock dependent systems such as The Netherlands (Central Bureau of Statistics The Netherlands, 2019). This was reflected in the stakeholders discussions with timid references to *“more sustainable stocking, which might mean a reduction in stocking rates”* (10) and *“re-distribution and smart live-stocking”* (101) (although actual destocking did get mentioned – see further discussion).

Still, some stakeholders did engage to a certain extent with such “deeper levers” of intent and design for a more transformational food system re-orientation (Abson et al., 2017). These included the consideration of changes in food markets (2), and the creation of new P markets, including the need for products from food/crop waste (40, 80) and for recovered P (81). This also included the calls for changes in policy and legislation to support new processes (26) and new markets (110), and the creation of suitable regulatory frameworks (72, 73, 88, 111) and government innovation strategies (42). Deeper levers appeared in the generic calls for integrated approaches (112) and the build-up of opportunities to change people’s perception (89) and education strategies (41). For the most part, however, even these deeper levers remained within what we would consider second loop in our analytical framework, since they do not represent necessarily transformations of structures and regimes. A general theme across all scenarios was the perception that many of the tools and solutions to addressing the P issue in Northern Ireland already exist and are already available, but they are not implemented sufficiently or appropriately. This resonates with arguments that have been made more globally, with Reitzel et al. (2019) arguing that the global P challenge is in part due to poor uptake of advances, for example, in approaches to reduce losses of P from agriculture. There are many land management practices that could already be adopted and could improve the situation, such as widespread adoption of soil sampling and nutrient management planning, but there is not sufficient uptake from farmers. Generally, there seemed to be the impression that, despite uncertainty and lack of evidence in certain areas, barriers to improvement are more related to governance and collaboration aspects rather than on technological ones, in line with what found in a study for the Republic of Ireland (Macintosh et al., 2019).

We did find, nevertheless, a few of transformative third-loop propositions. For example, we find that, in the Northern Ireland context, the calls for full new land use strategies (75, 114, 117), with new types of food production, aquaculture and hydroponics (102) and involving land use changes in which bio-energy crops are planted in place of grassland to drawdown more soil P (55) as a transformative move forward. Other suggested alterations are changes in plant species and rotations for better acquisition of soil P (13), or even the suggestion to move to potato and cereal crops with the intention to conserve legacy P in the soil (16) and the controversial destocking option (104). Re-imagined technological design included development of technology to export manures in a different way (14), pursuing methods to release higher amounts of organic P (15), new P recovery methods (54) and technology to support a mobile or centralized manure system (56). Strategic intent changes could involve development of integrated C, P and N solutions (116). More forward-thinking arguments include suggestions that P could be re-framed as a public good (105) and that consumer’s mind-set could be changed to recycle P (106), with the emergence of new P products (43).

The predominance of responses in the first and second loops over the triple-loop is aligned with findings from other – non P related - empirical studies exploring transformative potential through social learning processes with scenarios processes (Brown et al., 2016, Totin et al., 2018), and more generally (Kraker, 2017). It is possible that our own research design suffered from the same limitations that have been detected by Dorninger et al. (2020), who found that research methods and problem framing partially drive the type of interventions that emerge out of this kind of studies. We could have possibly enhanced triple-loop responses by presenting stakeholders with yet more radical scenarios, for example, one in which destocking was made mandatory or one by which the world population became vegan. We did include one scenario on change of diet (scenario 3), which could have possibly been more transformation-inducing, but which was voted out by workshop participants when narrowing down the discussion from five to three scenarios. Indeed, Scenario 5 *Balanced system* (which in a way is the most transformative of the three that did get discussed in the workshop) did prompt a reaction about the need of a *“new framework”* to allow the required changes to happen, demonstrating acknowledgement for the need of a change in framings, and could serve as a basis for exploring more transformative scenarios. In this respect, the stakeholders included in this process can be seeing as forming part of the so-called 'establishment', which might make them less inclined to contest it. Further, the visibility of the workshop format may have limited the kinds of responses or views that stakeholders felt comfortable providing due to the needs of professional discretion or hidden power relationships between stakeholders. Other methods, such as confidential interviews with each person might have yielded different responses, but at the expense of knowledge co-production inherent to facilitated workshops.

Finally, while a further step of this analysis could have involved reaching out again to the stakeholders with our classification in the three loops, this would require considerable effort to introduce and verify a common understanding among participants of the academic concept of loop learning with enough detail to allow them to classify and find agreement among their statements.

## 5 Conclusion

Global food security and healthy freshwater and coastal ecosystems are in jeopardy if no transformative actions to make Phosphorus use more efficient and sustainable are taken across the food, agriculture, waste and other sectors. In this global P challenge, system’s reorientation requires an examination of stakeholder views and of the potential to move away from the current ‘P hungry’ and wasteful model. This paper has advanced previous conceptual work by developing and analysing a P transdisciplinary scenario process, assessing stakeholders responses in their potential for transforming P use in the food system, using Northern Ireland as a case study.

The process was successful in triggering a broader conversation of the P challenge, providing a clearer view of the complexity of the food system as a whole and discussing possible strategies of increased P sustainability at the regional scale. For example, since the time of the workshop, initiatives have taken place to help accommodate the asymmetric nature of nutrient flows in the regional food system: the Ministry of Agriculture in the case study, DAERA, has launched a scheme to promote increasing the production of protein crops, to reduce imported feed requirements. If implemented, this could potentially contribute to ‘thinning’ the inward flow of phosphorus via feeds to livestock). In addition, there has been an expert report submitted to the DAERA minister, on the development of a circular bio-economy in Northern Ireland, with the finding of the P SFA helping to inform the recommendation in this report.

For the most part, however, stakeholder responses remained transitional or incremental, rather than being fundamentally transformative. Nevertheless, the process did unveil some deeper levers that could be acted upon. In this respect, our results can be conceptualized as a spectrum on a transformational pathway, starting from coping to adaptive and transformative responses, each with implications for addressing the P problem. Borrowing from the Three Horizons heuristic of pathways transformation (Fazey et al., 2020, Sharpe et al., 2016), the minimal approach (first loop, coping by tweaking the status quo) sees increased knowledge and training for farmers and other stakeholders about P management, as well as increases in measuring and monitoring soils, water and agriculture for P and related issues, to enable more targeted interventions and adjustments to agricultural practices (Jacobs et al., 2017). However, the costs, resistance from entrenched interests and uncertainty from knowledge gaps constrains deeper and more effective action. The hesitancy and limited intervention are unlikely to show improvements to the P problem other than providing better data on P dynamics through improved monitoring and minor tweaks in practice, but fundamentally remaining in the *current* system. Results from the second loop show a system adapting through transitional efforts and incrementalism, a *transitional* system. A “*most feasible”* scenario for P management in Northern Ireland emphasises market mechanisms and incentives, some agricultural practices changes such as *“smart stocking”*, investment in research and innovation, and adoption of better P stewardship practices and technologies where possible. This evolved system would be governed by a package of new regulation and innovation strategies. This scenario adjusts but does not fundamentally transform the structure of the agricultural system in Northern Ireland, which is livestock dominated, and crucially is unlikely to solve the problem. Incrementalism might also be crucially too slow to given the long time lags involved in the P dynamics in soil and water (Hamilton, 2015, Jarvie et al., 2013). The third loop (transformative paradigm shift) could be seen as a transformed *aspirational* system, less livestock dependent with more arable agricultural regime featuring cereals, potatoes, and industrial crops to reduce soil P in present-day grasslands and increase effective recycling of manures. This system would be supported by a centralised or mobile manure management system for directly exporting manures away from farms (and thus accession to soils and water) and by integrated nutrient and strategic land use management policies and legislation and complemented by consumer-side emphasis on sustainable P diets and goods.

Generally, our results would indicate that, despite uncertainty and lack of evidence in certain areas, barriers to improvement are more related to governance and collaboration rather than on technological aspects. While these result from a one-off interaction, these deeper levers could be worked upon in continued participatory processes or other forms of sustained stakeholder engagement, to further challenge existing social norms and structures with regards to P’s role in a more sustainable and resilient food system. Participatory stakeholder methods such as serious games, foresight, and backcasting currently applied to other challenges such as climate change, may serve as appropriate approaches (Andreotti et al., 2020, Guillen et al., 2021, Rumore et al., 2016).

Some of the specificities of the discussion of our study of Northern Ireland may be of relevance to other livestock-dominated systems. More generally, though, the process itself can be expected to be useful more generally. Replicated and adapted elsewhere, this process can serve as diagnostics, as well as for the identification of those deeper levers, opening up avenues to work upon for global scale transformation.

## Declaration of Competing Interest

The authors declare that they have no known competing financial interests or personal relationships that could have appeared to influence the work reported in this paper.

## Footnotes

1 Constructivism is a post-positivist approach to scientific inquiry that explores ‘locally and specific constructed realities’, using dialectical processes to create consensus-based research results (Guba et al., 2018). In keeping with constructivism, our workshops, as dialectical occasions, sought to distil diverse participant views to identify learning and transformational potential for the local reality of Northern Ireland’s phosphorus management.

2 In fact, 39% of Northern Ireland’s waterbodies are failing the targets required for good water quality due to elevated P levels and the majority of these are located in areas of high agricultural intensity ().

3 The Ulster Farmers Union (Table 2) claims ‘over 11,500 members and their families’ against a total number of farmers in Northern Ireland between ± 17 000 and 25 000 depending on the source. Obviously, they do not capture all farmers and their views, but are the major farmer representative and advocacy organisation of influence in Northern Ireland, and thus the key, member-driven, farmer stakeholder. https://www.ufuni.org/membership.

4 Despite best efforts, some sectors still remained underrepresented, particularly the fertiliser and food and drink sectors. Government agencies might be seen to be overrepresented in our sample, but it should be noted that they span a large range of varied policy sectors.

5 They were explicitly asked not to vote for the ones that they considered most likely, but the ones that they found of most interest.

6 The reflections form was labelled ‘What has changed for you today?’, and included question such as: what did you expect from today’s discussions? have your views of Northern Ireland’s food system change? have you been surprised by any of the things that have come up in today’s discussions?, has anything from today’s discussion made you think differently about the role and views of stakeholders in Northern Ireland’s food system? and do you think there is anything that you or your organization should do differently?.

7 The team was composed of nine members from a variety of disciplines from soil, water and agricultural sciences, to social sciences and economics, with varying levels of familiarity with Northern Ireland’s food system, which allowed for a good balance between deep knowledge and external “fresh” views.
