# Supplementary material for: Are stakeholders ready to transform phosphorus use in food systems? A transdisciplinary study in a livestock intensive system
Source: Environ Sci Policy. 2022 May;131:177–87. doi: 10.1016/j.envsci.2022.01.011 (PMC8895547; doi:10.1016/j.envsci.2022.01.011)
Supplement: Supplementary file 1 — Supplementary material. [file mmc1.pptx]

## Slide 1
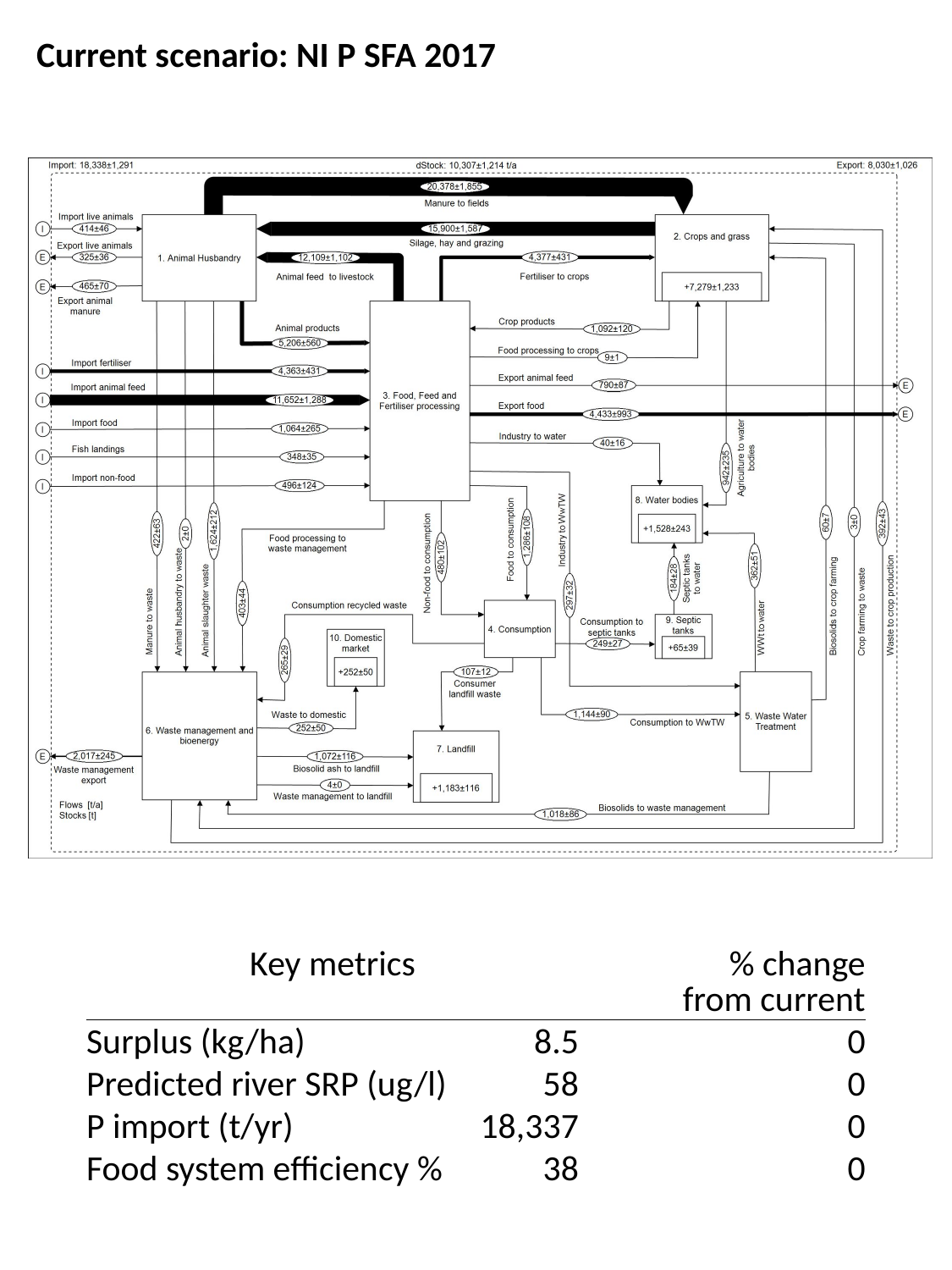

Current scenario: NI P SFA 2017
| Key metrics | | % change from current |
| --- | --- | --- |
| Surplus (kg/ha) | 8.5 | 0 |
| Predicted river SRP (ug/l) | 58 | 0 |
| P import (t/yr) | 18,337 | 0 |
| Food system efficiency % | 38 | 0 |

## Slide 2
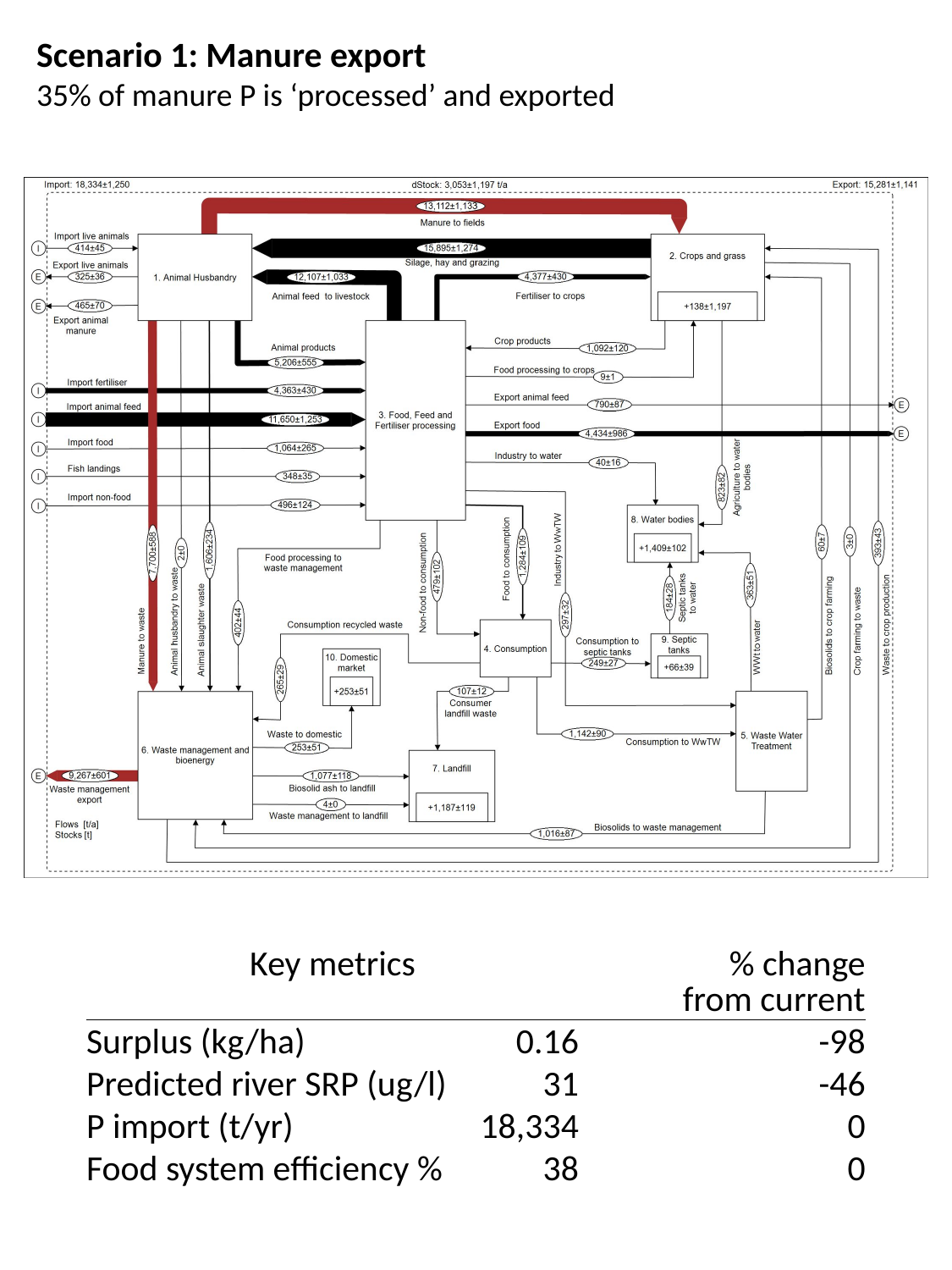

Scenario 1: Manure export
35% of manure P is ‘processed’ and exported
| Key metrics | | % change from current |
| --- | --- | --- |
| Surplus (kg/ha) | 0.16 | -98 |
| Predicted river SRP (ug/l) | 31 | -46 |
| P import (t/yr) | 18,334 | 0 |
| Food system efficiency % | 38 | 0 |

## Slide 3
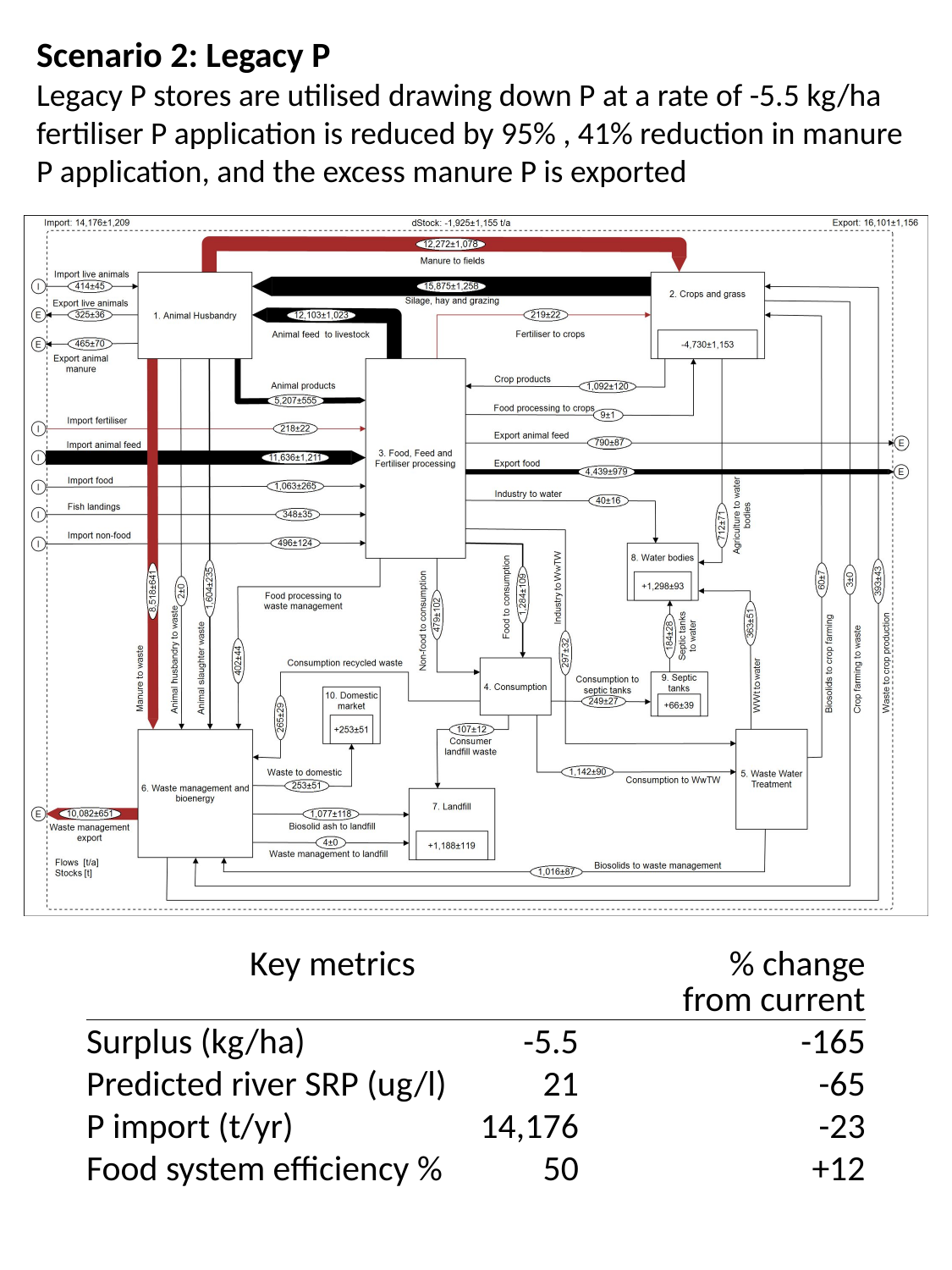

Scenario 2: Legacy P
Legacy P stores are utilised drawing down P at a rate of -5.5 kg/ha fertiliser P application is reduced by 95% , 41% reduction in manure P application, and the excess manure P is exported
| Key metrics | | % change from current |
| --- | --- | --- |
| Surplus (kg/ha) | -5.5 | -165 |
| Predicted river SRP (ug/l) | 21 | -65 |
| P import (t/yr) | 14,176 | -23 |
| Food system efficiency % | 50 | +12 |

## Slide 4
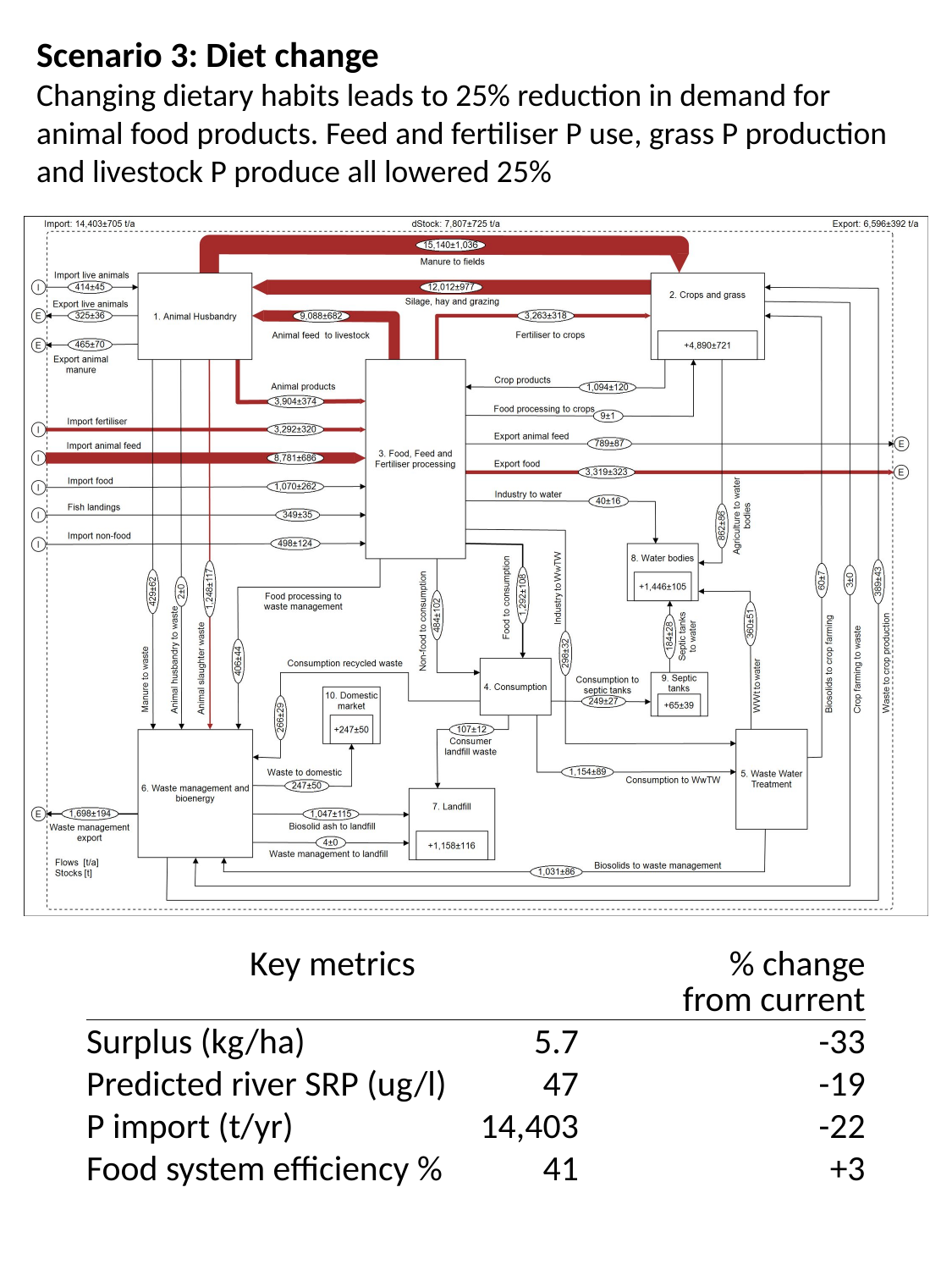

Scenario 3: Diet change
Changing dietary habits leads to 25% reduction in demand for animal food products. Feed and fertiliser P use, grass P production and livestock P produce all lowered 25%
| Key metrics | | % change from current |
| --- | --- | --- |
| Surplus (kg/ha) | 5.7 | -33 |
| Predicted river SRP (ug/l) | 47 | -19 |
| P import (t/yr) | 14,403 | -22 |
| Food system efficiency % | 41 | +3 |

## Slide 5
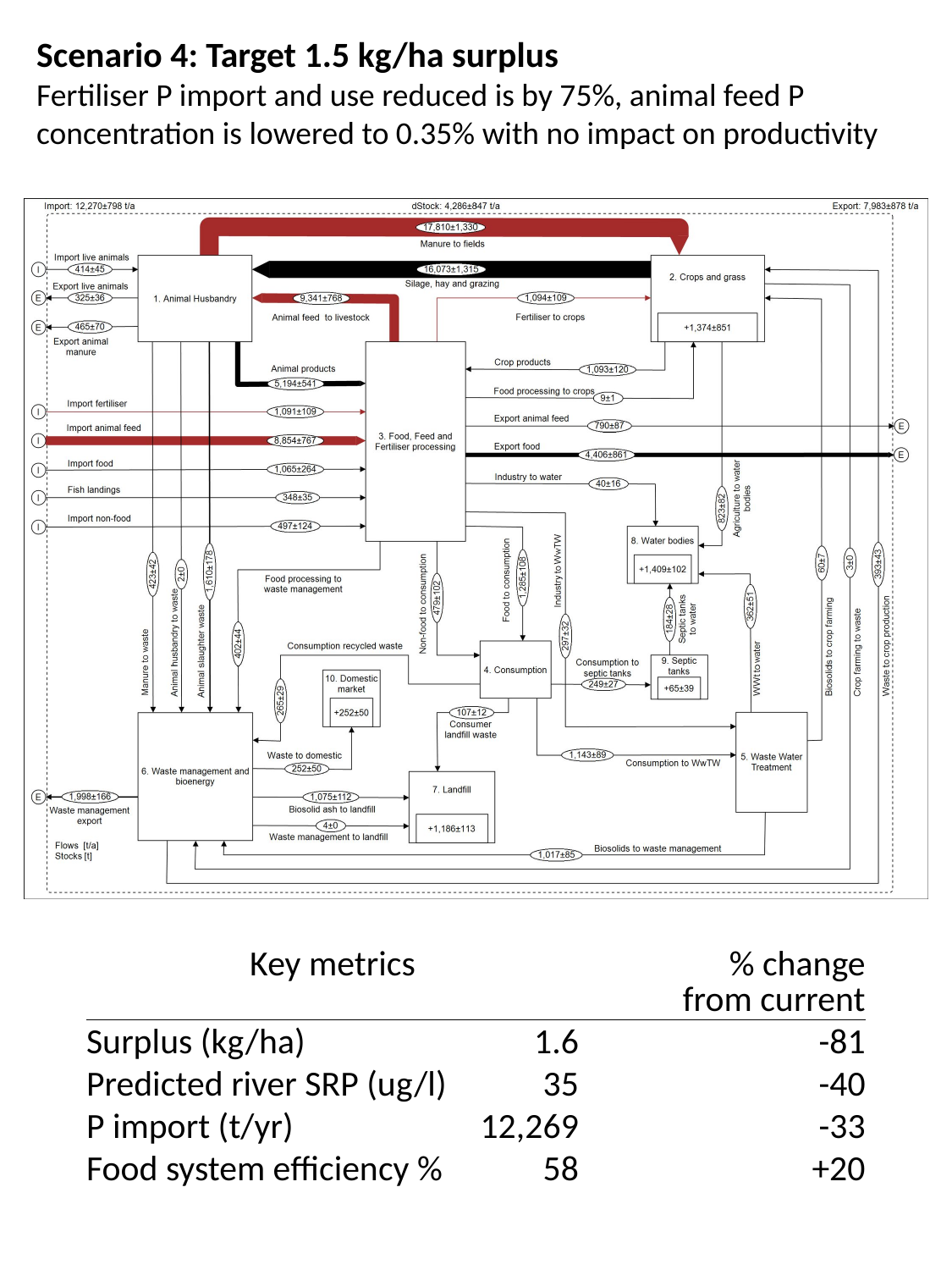

Scenario 4: Target 1.5 kg/ha surplus
Fertiliser P import and use reduced is by 75%, animal feed P concentration is lowered to 0.35% with no impact on productivity
| Key metrics | | % change from current |
| --- | --- | --- |
| Surplus (kg/ha) | 1.6 | -81 |
| Predicted river SRP (ug/l) | 35 | -40 |
| P import (t/yr) | 12,269 | -33 |
| Food system efficiency % | 58 | +20 |

## Slide 6
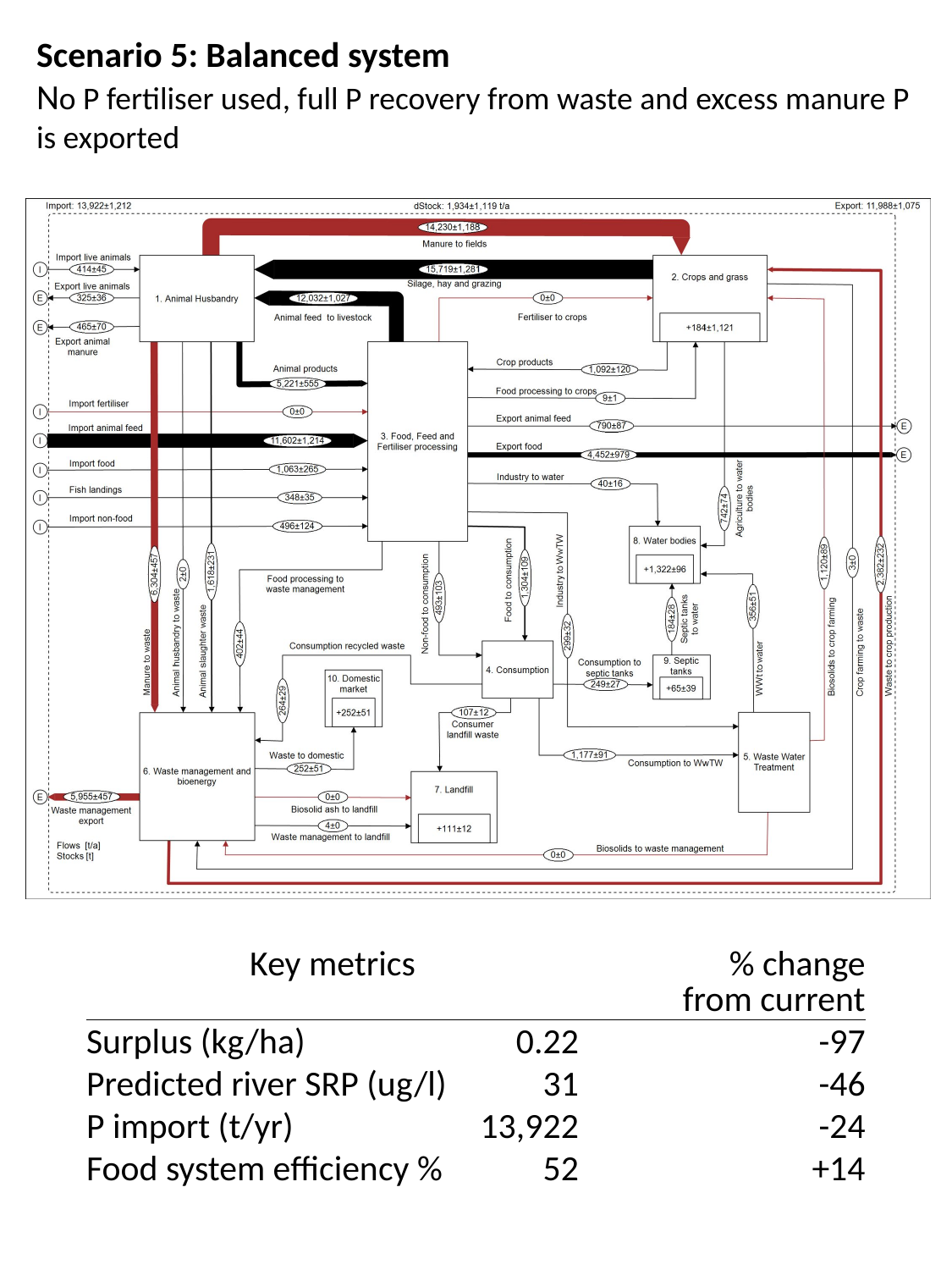

Scenario 5: Balanced system
No P fertiliser used, full P recovery from waste and excess manure P is exported
| Key metrics | | % change from current |
| --- | --- | --- |
| Surplus (kg/ha) | 0.22 | -97 |
| Predicted river SRP (ug/l) | 31 | -46 |
| P import (t/yr) | 13,922 | -24 |
| Food system efficiency % | 52 | +14 |
