# Supplementary material for: Are stakeholders ready to transform phosphorus use in food systems? A transdisciplinary study in a livestock intensive system
Source: Environ Sci Policy. 2022 May;131:177–87. doi: 10.1016/j.envsci.2022.01.011 (PMC8895547; doi:10.1016/j.envsci.2022.01.011)
Supplement: Supplementary file 2 — Supplementary material. [file mmc2.docx]

**Supplementary materials 2**

Tables 4, 5 and 6 present respectively the responses to the scenarios *Scenario 2 Legacy P, Scenario 4 – Target 1.5kg/ha* and *Scenario 5: Balanced System,* classified according to the three loops. Statements are numbered for ease of referencing, but numbers do not relate to any chronological or hierarchical ordering.

Table 4. Learning loops from Scenario 2: Legacy P

| **Domain** | **Single loop** | **Double loop** | **Triple loop** |
| --- | --- | --- | --- |
| Institutions |  | 1. Technologies for renewable energy e.g. Anaerobic Digestion (AD) 2. A change in future food markets – switch of products to suit new markets 3. Paying farmers for public goods |  |
| Norms | 1. Diversification to higher value products 2. Soil testing becoming compulsory Northern Ireland wide 3. Processes need to be introduced for targeted reductions in the right places | 1. Industry to realise there is an excess (this message needs to be brought to industry not just environment). 2. Manure is the wrong concept to look at – other ways? E.g. bio-char 3. Technology to support a circular bio-economy 4. More sustainable stocking – which may mean a reduction in stocking rates 5. Better soil management to make better use of the P in the soil 6. Diversion of manure flows to AD and energy production | 1. Change plant species and/or reseed rotations to identify those that can extract the most P 2. Technology use to enable exportation another way, valorisation by other means (regionally centralised approach) 3. Developing methods to release higher amounts of organic P 4. Move to cereal and potato crops which would conserve P |
| Actor Network | 1. Share/communicate evidence of P in soils 2. More producer groups |  |  |
| Multi-Level Interactions | 1. Improvements in knowledge transfer and research to engage and advise farmers | 1. Knowledge transfer, to support a different system | 1. New map framework needed to support redistribution |
| Governance | 1. Compliance and incentives 2. Enforcement 3. Policy changes targeted to priority area 4. More focused funding – with more integration | 1. Change in policy/legislation to support new processes |  |
| Uncertainty | 1. Too many unknowns will limit uptake 2. Major research gap in developing robust management guidelines 3. We don’t know how long this will need to happen for. Will this lead to a P deficit? 4. Not enough research and scientific evidence to support scenario 5. The completion of a wider cost benefit analysis 6. Too many unknowns (climate change, infrastructure needs, attitudes) 7. Difficulty in sourcing low P feed (e.g. for dairy sector) and costs of feed could increase as a result | 1. More research to support use of P in high P soils |  |

Table 5. Learning loops from Scenario 4: Target 1.5kg/ha

| **Domain** | **Single loop** | **Double loop** | **Triple loop** |
| --- | --- | --- | --- |
| Institutions | 1. Incentives 2. No major restructuring required 3. Feed formulation | 1. Creation of new P markets 2. New education, strategy and policy needed 3. DAERA science innovation strategy | 1. Creation of new P products |
| Norms | 1. Up-to-date analysis of forage, grazing, etc. 2. Education/training for P 3. Distribution of manures are currently uncontrolled/inefficient 4. Improvements in grassland management so less dependence on feed 5. Reduction in chemical P use | 1. More manure processing to increase P availability when recycled 2. Optimise P retention in the system 3. Mind-set of slurry as a resource vs waste 4. The feed sector will need to source low P ingredients 5. System’s actors willingness to change | 1. New P recovery methods utilised 2. Potential land use change e.g. bio-energy crops such as hemp replacing grassland to drawdown more soil P 3. Technology to support a mobile or centralised manure system |
| Actor Network | 1. There will be lobbying against the changes to the fertiliser sector and prices may go up 2. Get the message to stakeholders/farmers, online and advisors (agronomists) 3. De-watering technology is labour intensive and need to think of economies of scale, farm size (small farms) | 1. Potentially could economically benefit farmers due to less fertiliser costs 2. Water industry resources diverted from addressing P pollution could be applied elsewhere 3. Technology for manure and P processing – system or process for better redistribution. 4. Industry/SME groups /collaborative networks for nutrient management, tech development and implementation 5. Changes in the next generation of agronomists 6. Recovery of the dairy/AD sector – increased development of AD facilities 7. Utilisation of AD plants as a resource 8. Precision diet nutrition | 1. Fertiliser sector will have to rebrand to suit changed demand |
| Multi-Level Interactions | 1. More accurate targeting and stakeholder interaction 2. Optimising the diverse system 3. Knowledge transfer: this is important because a lot is based on tradition |  |  |
| Governance |  | 1. Regulatory framework needed 2. New regulation and enforcement 3. Innovation strategy | 1. A new land use strategy |
| Uncertainty | 1. Cost of concentrate may go up which impacts the feed sector 2. Lack of knowledge – information sources | 1. More research and education surrounding implementing the changes required 2. Slurry/nutrient management. Technologies exist but what technology? At what scale? What markets (fertiliser, ash, biochar, fuel, other value engineered compounds)? |  |

Table 6. Learning loops from Scenario 5: Balanced system

| **Domain** | **Single loop** | **Double loop** | **Triple loop** |
| --- | --- | --- | --- |
| Institutions |  | 1. New products of food/crop waste. Needs new markets, opportunities e.g. digestate 2. Create a market for recovered P 3. New product development, due to the new source of P, blends and products |  |
| Norms | 1. How to make the needed changes simple and enough for the farmers? 2. Increases monitoring and management e.g. soil testing across soil types 3. Metrics need to be identified to communicate surplus and monitor 4. Targeted interventions 5. Optimise P in the grass in Northern Ireland | 1. Standardisation and a framework for P manure management 2. Building opportunities to change people’s perceptions of the system 3. Re-design/re-engineering of waste-water treatment (incinerating sludge process, economically viable P recovery and redesign) 4. Change to food and crop waste processing 5. Recover P (manure P) 6. Cut animal feed imports 7. Opportunities for the AD sector 8. Re-design food process (technology) 9. New approaches to monitoring and measuring 10. Transforming materials into inorganic P fertiliser 11. Diversity into other areas through advisory services 12. To value P self-sufficient agricultural production 13. Bio-solid to grassland versus crop 14. Re-distribution and smart live stocking (less concentration, avoidance next to rivers) | 1. New food production, more emphasis on aquaculture, hydroponics, etc. 2. Blending recycled P 3. Destocking 4. Recovered P promoted as a public good |
| Actor Network |  |  | 1. Change to consumer mind-set to recycle P |
| Multi-Level Interactions | 1. More accurate targeting and stakeholder interaction |  |  |
| Governance | 1. Agri-production to continue 2. Increased investment in agri-industry | 1. Regulations to allow for new markets 2. Establish regulation framework for stakeholders 3. Integrated approach 4. Need to rethink pasture-land fragmentation and land use | 1. Land use change – Adoption of hydroponic within the agri-food industry; arable. 2. Radical thinking 3. Integrated solutions for C, P and N 4. Development of a land management strategy |
| Uncertainty | 1. Practical barriers to the recovery of P | 1. Slurry/nutrient management. Technologies exist but what technology? at what scale? what markets (fertiliser, bio-chard, fuel)? 2. Which is the right metric to use to monitor and communicate? 3. Is there a demand outside Northern Ireland for P exports? |  |
